# Multimodal operando characterization unravels polaron accumulation and ion dynamics in high-stability ambipolar OECTs

**DOI:** 10.1126/sciadv.aea9786

**Published:** 2026-04-15

**Authors:** Haoyu Zheng, Ruizhe Wang, Hengyi Ma, Xiang Li, Gang Ye, Yuanchun Zhao, Ping Zhang, Gang Wang, Hengda Sun, Yongri Liang, Simone Fabiano, Kai Xu

**Affiliations:** ^1^ Center for Extreme Deformation Research, State Key Laboratory of Metastable Materials Science and Technology, Yanshan University, Qinhuangdao 066004, China.; ^2^ State Key Laboratory of Advanced Fiber Materials, College of Materials Science and Engineering, Donghua University, Shanghai 201620, China.; ^3^ Key Laboratory for the Green Preparation and Application of Functional Materials, Hubei Key Laboratory of Polymer Materials, School of Materials Science and Engineering, Hubei University, Youyi Road 368, Wuhan 430062, China.; ^4^ State Key Laboratory of Metastable Materials Science and Technology, Yanshan University, Qinhuangdao 066004, P. R. China.; ^5^ School of Electrical Engineering and Automation, Jiangxi University of Science and Technology, Ganzhou, Jiangxi 341000, China.; ^6^ Pingdingshan Industrial Technology Research Institute, Henan Academy of Sciences, Henan, Zhengzhou 450046, China.; ^7^ Laboratory of Organic Electronics, Department of Science and Technology, Linköping University, Norrköping SE-60174, Sweden.; ^8^ Wallenberg Wood Science Center, Department of Science and Technology, Linköping University, Norrköping SE-60174, Sweden.

## Abstract

Achieving both high performance and stability in ambipolar organic electrochemical transistors (OECTs) remains challenging, largely due to limited understanding of how polymer structure and doping mechanisms interplay. Addressing this requires operando techniques that capture both structural and charge dynamics. In this study, we use a multimodal operando approach to investigating P-6O, a naphthalenediimide and dialkoxybithiazole copolymer with oligo(ethylene glycol) side chains. Operando electron paramagnetic resonance (EPR) and x-ray photoelectron spectroscopy (XPS), supported by density functional theory, reveal the formation of localized p- and n-polarons due to low backbone planarity, a characteristic often considered a design challenge for achieving robust transport and stability. However, P-6O–based OECTs exhibit pulsing stability. attenuated total reflection Fourier transform infrared and temperature-dependent EPR indicate strong polymer-electrolyte interactions, while operando XPS and grazing incidence wide-angle x-ray scattering reveal bidirectional ion motion that preserves film morphology. Collectively, these results underscore the critical role of ion dynamics in stabilizing OECTs, even in less planar polymers, and offer design guidelines for stable ambipolar transport.

## Teaser

A balanced ion exchange in a conducting polymer film prevents degradation and boosts organic transistor stability.

## INTRODUCTION

Organic electrochemical transistors (OECTs) have emerged as promising candidates for applications in bioelectronics, sensors, and flexible electronics due to their unique ability to modulate electronic conductivity through ionic interactions (*1*–*4*). These devices operate by injecting ions from an electrolyte into a conducting polymer channel, thereby altering its electrical conductivity. Hence, conjugated polymers with mixed ionic-electronic transport capability have gained notable attention, enabling high-performance OECTs that operate at low voltages and exhibit high transconductance (*5*–*7*). Despite these advantages, ensuring device stability remains a challenge, as repeated doping and dedoping cycles in complex electrochemical environments can cause material degradation and compromise device performance. Therefore, understanding how these conjugated polymer systems behave under electrochemical doping is essential for further optimizing OECT devices.

Operando characterization techniques, which enable real-time monitoring of structural, electronic, and ionic changes during device operation, offer direct insights into the mechanisms that govern performance and, critically, long-term stability. For instance, Keene *et al.* (*8*) used operando optical microscopy to visualize electrochemical doping fronts in conjugated polymers, revealing that hole transport limitations at low doping levels slow the doping rate and response time. Paulsen *et al.* (*9*) used operando grazing incidence wide-angle x-ray scattering (GIWAXS) to uncover lamellar expansion, structural hysteresis, and irreversible swelling upon air exposure, factors directly linked to device instability. While these and other studies have used various operando techniques to identify individual factors influencing OECT performance and stability, a comprehensive understanding of the interplay between electronic state evolution (e.g., polaron dynamics), polymer-ion interactions, and morphological changes, particularly for ambipolar systems, remains elusive. The precise correlation between these multifaceted factors and their collective impact on the long-term operational stability of conjugated polymer OECTs is not yet fully established. Thus, a cross-validated, multimodal operando approach is essential to deconstruct these complex relationships and gain robust mechanistic insights into device stability, paving the way for targeted material and device design strategies.

In this study, we investigate the ambipolar polymer P-6O, a copolymer comprising naphthalenediimide (NDI) and dialkoxybithiazole (2Tz) units with oligo(ethylene glycol) (OEG) side chains, as an active material for OECTs to elucidate the charge transport mechanisms and ion dynamics during electrochemical doping using a suite of operando electrochemical characterization techniques, including absorption spectroscopy, electron paramagnetic resonance (EPR), GIWAXS, and x-ray photoelectron spectroscopy (XPS). The absorption and EPR results, combining with density functional theory (DFT) calculations, reveal polymer’s electronic states upon doping, showing the relative localization of polarons. Further temperature-dependent EPR and attenuated total reflection Fourier transform infrared (ATR-FTIR) spectroscopy confirm the interactions between ions and polymers, and the swelling of the polymer upon contact with the electrolyte further supports the strong interaction. Operando GIWAXS results demonstrate negligible swelling during electrochemical doping, suggesting morphological stability, which is believed to be crucial for device durability. In addition, operando XPS analyses provide detailed insights into the ion dynamics during doping, showing that both cations and anions move oppositely while maintaining the polymer volume unchanged. This work demonstrates that stable OECT operation can be achieved with a localized polaron structure exhibiting negligible swelling during doping, thereby addressing critical gaps in the field of OECT stability. Moreover, our multidisciplinary approach offers valuable insights for designing materials with enhanced performance and durability.

## RESULTS

**Fig. 1.**
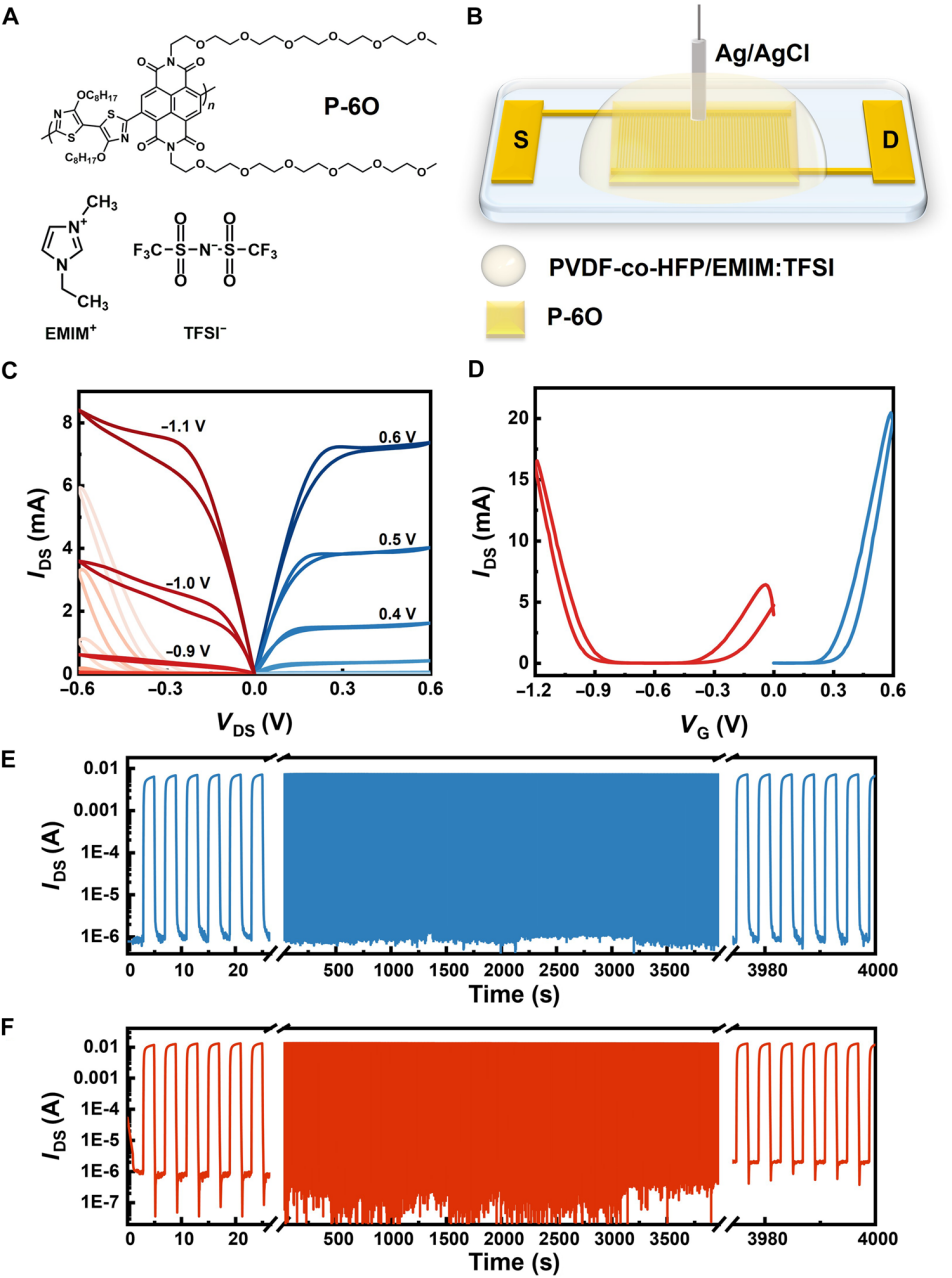
Electrical characterization of P-6O–based ambipolar OECT. (**A**) Chemical structures of P-6O. (**B**) Schematic drawing of the OECT structure. (**C**) Output curves and (**D**) transfer curves of the OECT. (**E**) A total of 1000 cycles of pulsing stability of the P-6O–based ambipolar OECT device. Under n-type operation, with *V*_DS_ = 0.1 V and *V*_G_ = 0 V (2 s) and 0.7 V (2 s); (**F**) under p-type operation, with *V*_DS_ = –0.4 V and *V*_G_ = –0.4 V (2 s) and −1.0 V (2 s).

Figure 1A illustrates the chemical structure of the ambipolar polymer, featuring NDI and 2Tz-based copolymers (P-6O). The OEG side chain, attached to the NDI unit, can improve ion mobility within the polymer matrix, leading to efficient mixed ion-electron conduction, which makes the polymer well suited for applications such as in OECTs.

First, the performance of the P-6O–based OECT was characterized. The device architecture is illustrated in Fig. 1B, where interdigitated gold electrodes with a channel length *L* = 5 μm and *W* = 238.5 mm. A 50-nm P-6O film was spin coated over the electrodes, followed by a dip-coated layer of polyvinylidene difluoride–co–hexafluoropropylene (PVDF-co-HFP)/1-ethyl-3-methylimidazolium bis(trifluoromethylsulfonyl)imide ([EMIM][TFSI]) gel electrolyte on top of the conducting polymer, and then a sintered Ag/AgCl pellet electrode was used as the gate electrode. The corresponding ambipolar transfer and output curves are given in Fig. 1 (C and D). The device, operating in accumulation mode, exhibits clear ambipolar characteristics with comparable output currents in both the p- and n-type regimes. The threshold voltage for hole transport (*V*_th_ = −0.8 V) is relatively higher than that for the electron transport (*V*_th_ = + 0.3 V), as shown in Fig. 1D. The extracted geometry-normalized transconductance (*g*_m,norm_) for the p- and n-type regimes are 0.34 and 0.42 S·cm^−1^, with C* value extracted from fig. S2 are 92 F·cm^−3^ for n-type at 0.7 V and 55 F·cm^−3^ for p-type at −1.1 V, corresponding to a μ value of about 0.030 cm^2^·V^−1^·s^−1^ for the n-type regime and 0.036 cm^2^·V^−1^·s^−1^ for the p-type regime (see also fig. S1) (*10*). Furthermore, we assessed its charge injection capability by applying a pulsed step voltage and integrating the gate current. The resulting charge density per unit volume at various voltages for both p- and n-type doping is shown in fig. S2. Values exceeding 10^20^ cm^−3^ were readily achieved, indicating a high doping level consistent with the electrochemical doping nature of the gating process. As shown in Fig. 1 (E and F), the polymer maintains a stable on/off current ratio (>10^3^) during continuous pulsing in both n- and p-type modes, with negligible degradation observed for 1000 pulse cycles. This is consistent with previous reports demonstrating its stability (*11*, *12*).

**Fig. 2.**
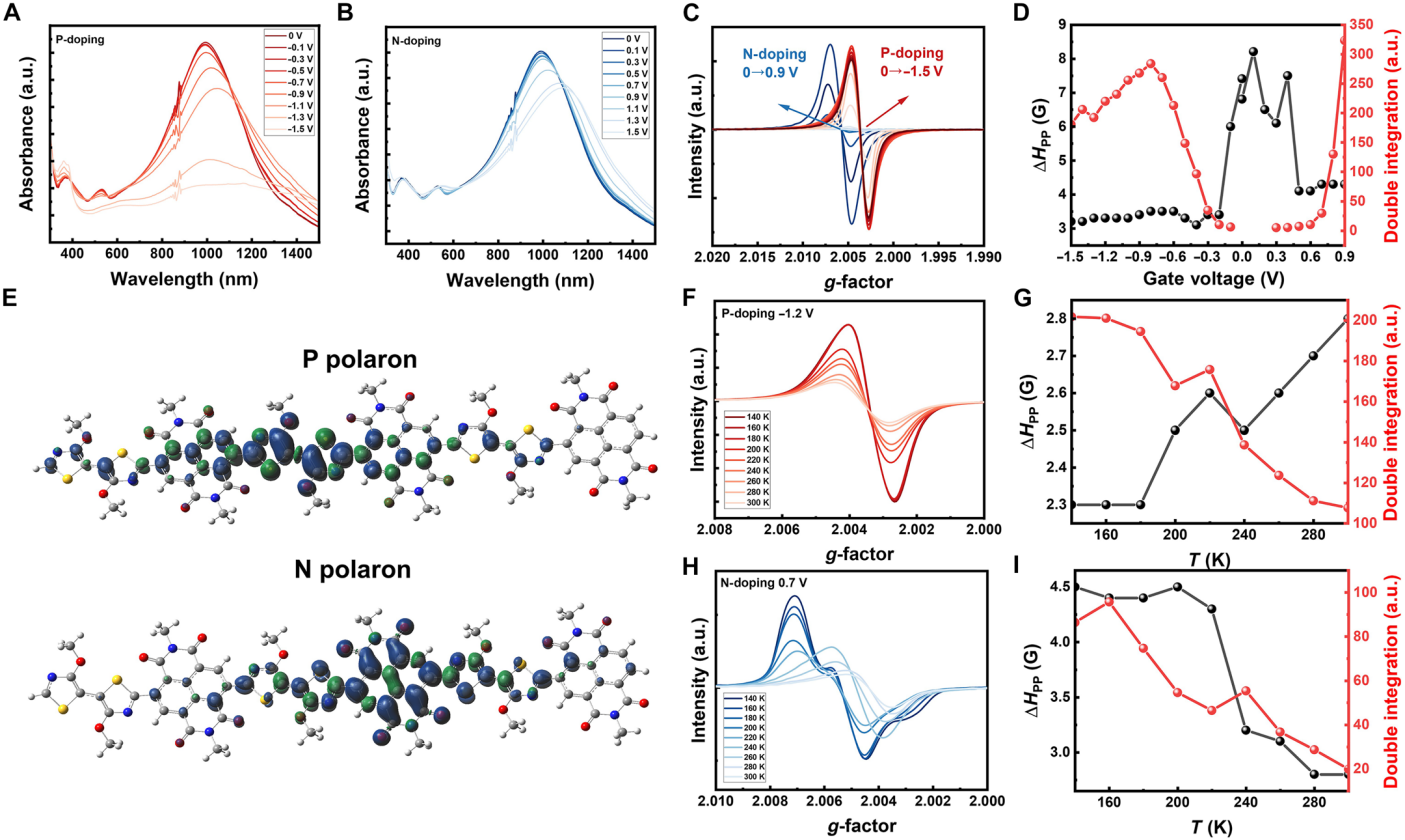
Characterization the n/p polarons of P-6O. (**A**) Electrochemical ultraviolet-visible-near infrared absorption spectra of p-type doping. a.u., arbitrary units. (**B**) Electrochemical UV-Vis-NIR absorption spectra of n-type doping. (**C**) Operando electrochemical EPR of P-6O; same device was used for n- and p-type doping. (**D**) The linewidth of EPR signal Δ*H*_PP_ and relative intensity change under different electrochemical doping voltages. (**E**) DFT calculated spin density map of n- and p-type doping at the ωB97XD/6-311G(d,p) level of theory. EPR signals of p polarons (**F**) and n polarons (**H**) at different temperature, corresponding peak-to-peak linewidth Δ*H*_PP_ and relative intensity change as a function of temperature of p polarons (**G**) and n polarons (**I**).

The oxidation/reduction of P-6O was confirmed using operando spectroelectrochemistry measurements. Polymer films were spin coated onto cleaned indium tin oxide (ITO) glass substrates and covered with a fast-annealed transparent PVDF-co-HFP/[EMIM][TFSI] electrolyte, connected to a sintered Ag/AgCl pellet electrode. The absorption characteristics under p- and n-doping conditions are presented in Fig. 2 (A and B), respectively. The neutral intramolecular charge transfer (ICT) absorption peak of P-6O is observed around 995 nm. Upon p-type doping (increasing oxidation potential), the ICT absorption gradually diminishes, while new features emerge at 363, 610, and 1200 to 1400 nm, which are likely associated with polaronic absorption and a potential weakening of ICT due to localized charges on P-6O. Similarly, under n-type doping (increasing reduction potential), the ICT absorption also decreases, accompanied by the appearance of a broad peak in the range of 1100 to 1500 nm and a distinct peak around 560 nm. These results indicate that both p- and n-type doping substantially modify the electronic states of P-6O, suppressing ICT transitions and generating new polaron absorption peaks (*13*, *14*).

Operando electrochemical EPR was used to study spin density and distribution during oxidation and reduction. For the experiment, a thin gold layer (~30 nm) was deposited onto a polyethylene terephthalate (PET) substrate (3 mm by 30 mm) to serve as source and drain electrodes, with a channel width of 0.5 mm. The polymer was then drop coated onto the substrate. The PVDF-co-HFP/[EMIM][TFSI] electrolyte was dip coated on top of the P-6O layer, and an Ag/AgCl paste electrode was connected to the electrolyte, positioned outside the EPR resonant chamber. The setup is illustrated in fig. S3A, and the same device was used for both oxidation and reduction measurements. Because P-6O is more stable when n-doped than p-doped, we first performed operando EPR under n-type electrochemical doping, followed by p-type doping. The gate and drain currents recorded during the EPR tests are shown in fig. S3B.

The EPR spectra, shown in Fig. 2C, reveal distinct behaviors for p-and n-type doping. For p-type doping, a clear spin-active (polaron) signal starts at *V*_G_ = −0.3 V, with maximum intensity observed at −0.8 V. As the gate voltage increases further, the signal gradually decreases, possibly due to the formation of multiply-charged species (e.g., bipolarons). In contrast, during n-type doping, the spin-active signal emerges at *V*_G_ = 0.3 V and increases sharply between 0.6 and 0.9 V. However, the *g*-factors for p-and n-type polarons differ: The *g*-factor for n-type polarons is 2.0051, consistent with other n-doped NDI-based polymers (*15*), while the *g*-factor for p-type polarons is slightly lower at 2.0036. The *g*-factor is a fundamental parameter in EPR that reflects the intrinsic electronic structure of an unpaired electron. Even seemingly small shifts (on the order of 10^−3^) are well above the instrumental resolution and correspond to differences in spin-orbit coupling and radical localization. This difference suggests distinct magnetic environments, implying that the n- and p-type polarons are relatively localized on the NDI or 2Tz units, respectively. To further support this interpretation, we performed DFT calculations on oligomers consisting of three repeating NDI-2Tz units. The optimized structures showed reduced planarity upon electron addition (n-type) or removal (p-type). The calculated spin density distributions, shown in Fig. 2E, indicate that for the p-type doping, the spin density is mainly localized on the 2Tz moiety with slight extension to adjacent NDI units. In contrast, for n-type doping, the spin density is predominantly located on the NDI unit, with minimal contribution from the neighboring 2Tz group.

The variation in linewidth can provide insight into the relative localization of polarons, as polaron localization affects spin motion narrowing and spin diffusion (*16*–*18*). The peak-to-peak linewidth (Δ*H*_PP_), shown in Fig. 2D, exhibits a broad signal in the voltage range of −0.3 to 0.4 V, which is likely due to trapped charges with strong localization. As the gate voltage increases beyond this range, Δ*H*_PP_ remains relatively stable, suggesting that significant polaron delocalization does not occur even at higher voltages.

Temperature-dependent EPR measurements were performed on the same samples under n-type electrochemical doping at 0.7 V and p-type doping at −1.2 V, with *V*_DS_ = ±0.1 V. Unlike typical conducting polymers doped with molecular dopants, where decreasing temperature gradually leads to linewidth broadening due to enhanced localization (*16*, *19*, *20*), our electrochemically doped system is compensated by mobile ions. In the anhydrous ionic liquid, ion motion is not screened by water and can directly interact with the polymer (*21*). Consequently, the temperature-dependent EPR line shape reflects not only intrinsic polaron localization but also the evolving ionic microenvironment surrounding the polaron.

The results are shown in Fig. 2 (F to I), we observed that under p-type doping, the linewidth increases with rising temperature. In addition, the signal intensity drops sharply around 180 K, accompanied by a small shift in the *g*-factor. For n-type doping, the signal intensity also decreases significantly around 180 K, correlating well with the changes in gate current observed during EPR tests (fig. S4). Specifically, the gate current becomes measurable above 180 K, indicating that ions start to move. Further differential scanning calorimetry measurements of the [EMIM][TFSI] gel (fig. S5) shown a *T*_g_ of 185 K (*22*), suggesting that ion dynamics may strongly influence the polarons and point to an interaction between the electrolyte and P-6O. In addition, for n-type doping, the EPR line shape exhibits significant temperature dependence. As shown in Fig. 2H and fig. S6, the EPR spectrum at 140 K displays clear splitting into two signal components, arising from frozen ion-polymer environments below *T*g. At higher temperatures, rapid ionic reorganization dynamically averages these environments, yielding a single unsplit line without altering the *g-*value. Overall, the temperature-dependent EPR spectra provide direct microscopic evidence that polarons in P-6O are embedded in and strongly modulated by an ion-defined local electrostatic environment.

**Fig. 3.**
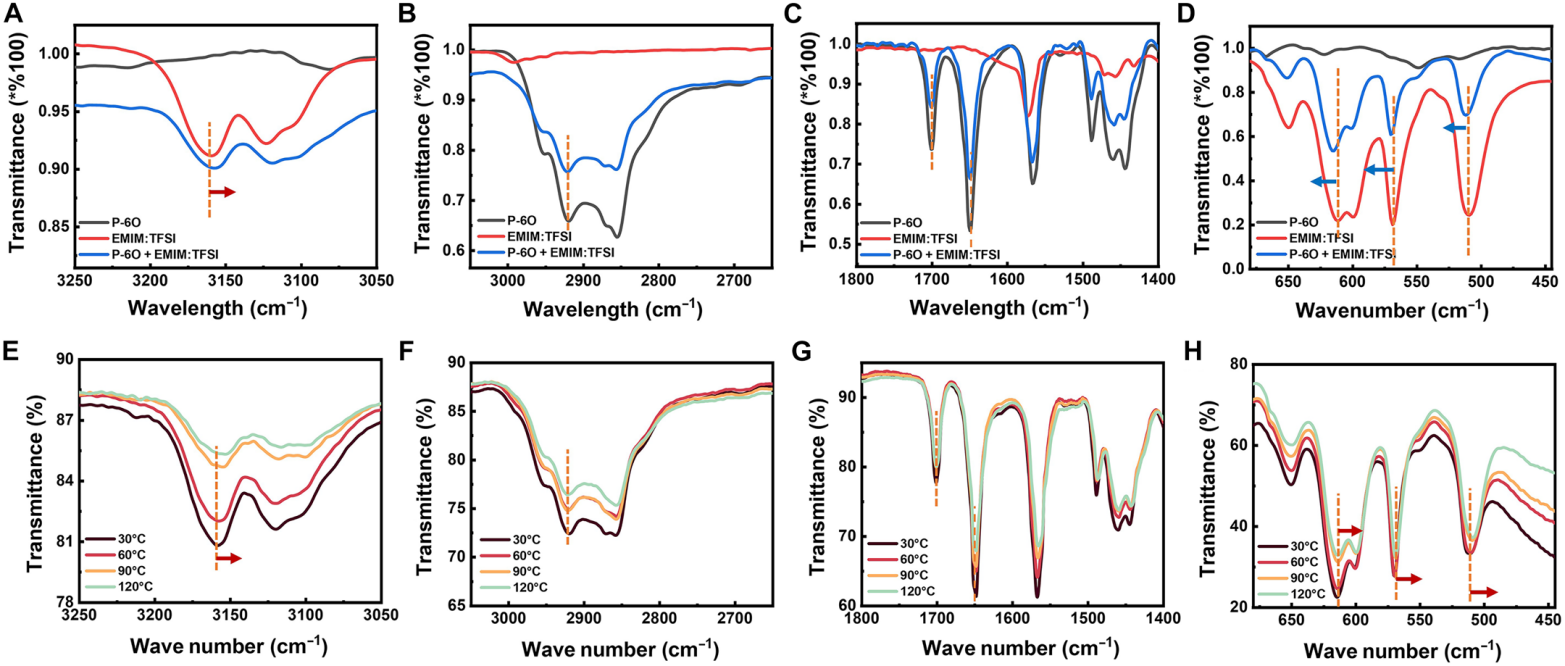
Confirmation of polymer-electrolyte interaction by ATR-FTIR. Different FTIR regions (**A** to **D**) of [EMIM][TFSI]. Different FTIR regions (**E** to **H**) of P-6O film after in contact with [EMIM][TFSI] under different temperature.

We further confirmed the polymer-electrolyte interaction using ATR-FTIR by measuring the spectra of the [EMIM][TFSI] electrolyte, pristine P-6O film, and the P-6O film after contact with the electrolyte (composite film) with full spectrum shown in fig. S9A. By comparing the differences among the spectra, we divided the analysis into four regions, as shown in Fig. 3 (A to D). The C─H vibration band at 3100 to 3200 cm^−1^ (Fig. 3A) is attributed to the [EMIM] cations, as this peak is absent in the pristine P-6O spectrum. A red shift of this peak in the composite film indicates that the C─H stretching modes of EMIM^+^ become slightly weakened upon interaction with P-6O, consistent with nonspecific ion-dipole or C─H···π interactions between the cation and the polymer. The C─H vibrations in the 2800- to 3000-cm^−1^ range are assigned to the methyl and methylene groups on P-6O; here, we observed negligible changes between the pristine P-6O and the composite film. The region from 1800 to 1400 cm^−1^ (Fig. 3C) includes peaks at 1700 and 1648 cm^−1^, likely corresponding to C═O stretching from the imide group (*23*), which also remained unchanged in the composite film. The range of 900 to 1400 cm^−1^ (fig. S9B) shows overlapping vibrations from both [EMIM][TFSI] and P-6O, making it difficult to assign individual contributions. These unshifted backbone signatures indicates that [EMIM][TFSI] does not form chemical bonds with P-6O but instead interacts via weaker noncovalent forces. Last, Fig. 3D highlights the 500- to 650-cm^−1^ region, which can be attributed to the asymmetric bending of -CF_3_, -SO_2_, or S─N─S groups (*24*). In the composite film, these vibrations exhibit a clear blue shift relative to pristine [EMIM][TFSI], indicating a slight stiffening of these internal modes. This stiffening is consistent with a modest withdrawal of electron density or a more constrained local environment when TFSI^−^ interacts with positively polarized segments of P-6O. The results are consistent with the well-known softness and delocalized charge of TFSI^−^, which promotes noncovalent ion-dipole interactions with π-conjugated polymer, aligning with general trends reported for conducting polymer/ionic liquid pair (*25*), where soft anions such as TFSI^−^ interact much more weakly while hard anions such as Cl^−^ induce stronger perturbations or even redox reactions. To distinguish between specific and general interactions, we compared ATR-FTIR spectra of P-6O after contact with LiTFSI, [EMIM][Cl], [EMIM][BF_4_], and [EMIM][OTf] in fig. S7. Only [Li][TFSI] and [EMIM][Cl] caused measurable shifts in P-6O backbone vibrations (e.g., peaks at 1700 and 1648 cm^−1^), suggesting stronger coordination or partial complexation likely due to high charge density of Li^+^ and the hard-base character of Cl^−^. In contrast, [EMIM][TFSI], [EMIM][BF_4_], and [EMIM][OTf] induced negligible backbone spectral changes, indicating weak nonspecific interactions. In addition, the broadening of the P-6O optical absorption spectrum after contact with [EMIM][TFSI] (fig. S8), rather than a peak shift typically observed during electrochemical redox reactions, is analogous to previously reported IL-induced structural disorder and chain-packing perturbations in poly(3,4-ethylenedioxythiophene)-based systems (*26*) and further indicates the weak noncovalent ion-dipole interactions. In summary, the ATR-FTIR results confirm weak noncovalent ion-dipole interactions between P-6O and [EMIM][TFSI], as shown by the shift in C-H and -CF_3_, -SO_2_, and S─N─S vibrational modes. Temperature-dependent FTIR further indicates that these interactions weaken at high temperature (*27*), with [EMIM][TFSI] vibrations shifting (Fig. 3, E and H) while those of P-6O remain unchanged (Fig. 3, F and G). Our results, when compared with other ionic liquid salts, indicate that soft anions cause only weak, nonspecific interactions, while hard ions generate stronger and measurable perturbations to the polymer backbone. This interaction is consistent with trends reported for other mixed ionic-electronic polymers, indicating that our mechanistic conclusions may extend beyond P-6O itself.

**Fig. 4.**
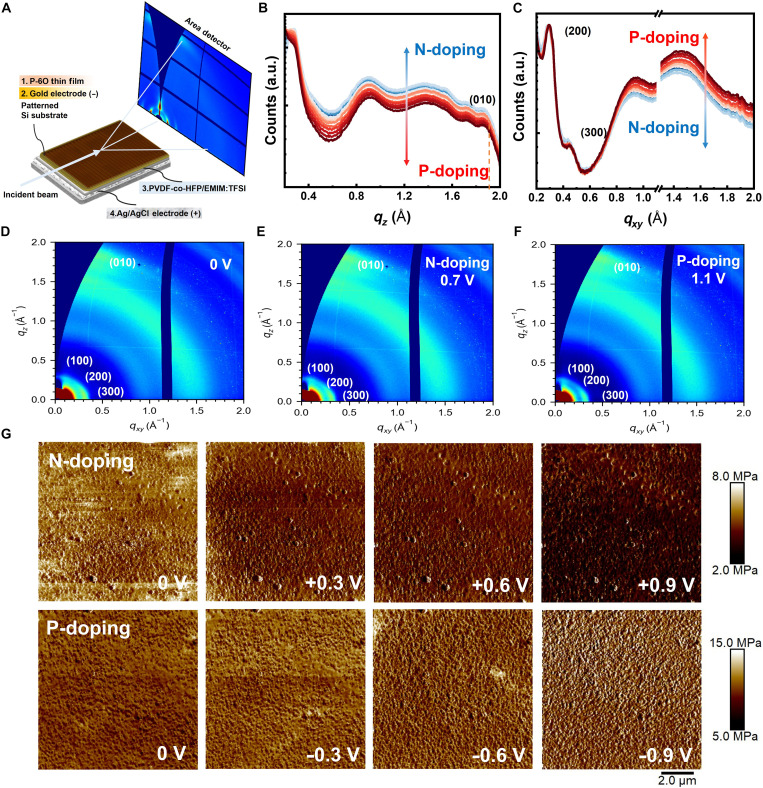
Characterizations of film morphology by operando GIWAXS and operando elastic modulus atomic force microscopy. (**A**) Schematic of the setup for operando GIWAXS. (**B**) Out-of-plane and (**C**) in-plane 1D line cuts of P-6O under different n- and p-type doping. 2D GIWAXS pattern of P-6O in contact with electrolyte (**D**), under n-type doping (**E**) and under p-type doping (**F**). (**G**) Operando atomic force microscopy (AFM) elastic modulus map of the polymer film under n- and p-type doping.

To further investigate the film morphology before and during electrochemical doping, we performed operando GIWAXS on the developed devices, as schematically illustrated in Fig. 4A. The polymer was laminated onto a substrate with etched through-channels (∼30 μm long, 20 mm wide, and spaced 200 μm apart), allowing ions from below to penetrate the top polymer film during doping (*28*). The film morphology was first performed under passive swelling conditions, with the polymer simply in contact with the electrolyte but without any applied bias, with ions permeate the P-6O film through the etched through-channels, allowing equilibration of the polymer with the electrolyte in the absence of charge injection. The two-dimensional (2D) GIWAXS pattern of the P-6O film in contact with the electrolyte (Fig. 4D) reveals a dominant face-on orientation (*29*). The π-π stacking distance slightly shifts from 1.82 to 1.86 Å^−1^, corresponding to a 2.03% decrease in π-π stacking distance from 3.45 to 3.38 Å, while the second-order lamellar diffraction (200) shifts from 0.40 Å^−1^ (pristine; fig. S10) to 0.29 Å^−1^, indicating a ∼38% increase in lamellar spacing from 31.40 to 43.31 Å. These changes occur before electrochemical doping is applied, indicating that the P-6O film undergoes passive swelling upon contact with the electrolyte.

For electrochemical d4oping, the device was stabilized for 2 min before each measurement, with a 30-s exposure time. The resulting 1D linecuts for n- and p-type electrochemical doping are shown in Fig. 4 (B and C), while typical 2D GIWAXS patterns at 0.7 V (n-doping) and −1.1 V (p-doping) are shown in Fig. 4 (E and F). We observed that during both doping types, the π-π stacking (010) distance remained unchanged, and the lamellar distance (200) shifted slightly (∼0.01 Å^−1^), much less than reported for other materials (*30*, *31*). This minimal swelling suggests a quasi–cross-linking effect, likely contributing to the high stability of the OECT by limiting structural changes (*2*). Figure 4G presents the atomic force microscopy (AFM) PeakForce QNM elastic modulus maps obtained under n-and p-type electrochemical doping. Under n-type doping, the modulus domains gradually decrease from 5.7 to 3.3 MPa at 1.2 V, while the surface roughness extracted from the corresponding height maps (fig. S12) shows no appreciable evolution. In contrast, under p-type doping, the modulus gradually increases, and the nanoscale domains become less defined at −1.2 V (fig. S13), reflecting the formation of a more constrained, anion-rich interfacial layer. This apparent mechanical stiffening is not accompanied by measurable geometric or volumetric changes: AFM height maps show only marginal variations in surface roughness (Δ*R*_q_ < 3 nm) (fig. S14). Operando Electrochemical Quartz Crystal Microbalance with Dissipation monitoring (EQCM-D) measurements (fig. S15) reveal negligible mass uptake during n-type doping and <1% mass uptake even at p-doping. Together, these results indicate that the observed modulus increase possibly originates from electrostatic and ion-polymer interactions within a thin interfacial region rather than from swelling or topographical artifacts, consistent with the volume-preserving mechanism inferred from GIWAXS.

XPS is widely used to identify elements and analyze oxidation states in conducting polymers, offering key insights into doping mechanisms and ion dynamics during electrochemical doping. Here, we performed operando electrochemical XPS using the same device structure as in the GIWAXS experiments but with a reduced device size (∼1 cm by 1 cm). The full XPS spectra under doping conditions are shown in fig. S17. In addition, we set 284.80 eV as the alpha carbon for reference baseline. Figure S16 presents the pristine XPS spectra of P-6O and [EMIM][TFSI], as we noticed the carbon signal at 286.68 eV possibly from the C─O group on P-6O, and two nitrogen signals were observed: one at 398.60 eV from N on Tz group, and 400.30 eV from N on imide group.

**Fig. 5.**
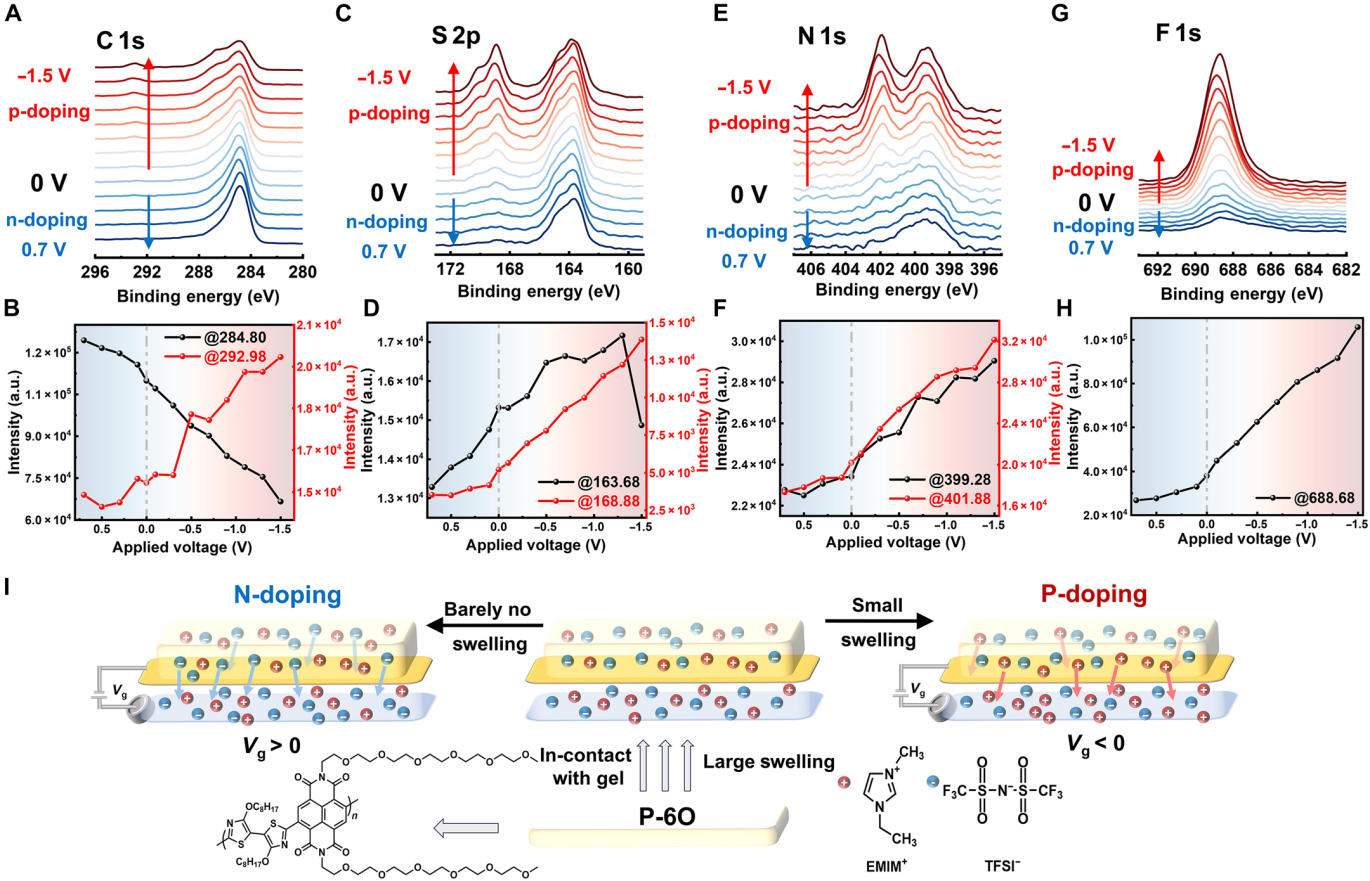
Characterizations of ions dynamics during n/p-type electrochemical doping by operando electrochemical XPS spectra. (**A**, **C**, **E**, and **G**) Detailed C 1s, S 2p, N 1s and F 1s spectra under n/p-type electrochemical doping, with corresponding peak intensities (**B**, **D**, **F**, and **H**) extracted from the spectra, respectively; the dashed green and red shaded areas are provided for guidance, representing n- and p-type doping, respectively. (**I**) Schematic of polymer film swelling upon contact with ion gel and ion dynamics during n/p-type electrochemical doping.

Figure 5A presents the C 1s spectra under electrochemical doping, During n-type doping, this peak shifts to lower binding energy, likely due to electron localization on the large conjugated NDI units. Under p-type doping, a new peak emerges at 286.68 eV, coresponding to carbon atoms in P-6O, indicating the increase of the P-6O proportion. In addition, the peak at 292.98 eV corresponds to carbon from the CF_3_ group in the [TFSI] anion and also indicates the increase of TFSI proportion. As shown in Fig. 5B, its intensity increases from n- to p-type doping, indicating that [TFSI] anion portion decreases during n-doping and increases during p-type doping.

Figure 5C shows the S 2p spectra under electrochemical doping, with two distinct peaks: one at 168.88 eV from sulfur in the [TFSI] anions and another at 163.68 eV from sulfur in the 2Tz group. The 168.88-eV peak increases steadily from n- to p-type doping, mirroring the trend seen for carbon in the -CF_3_ group. Similarly, the N 1s (399.28 eV; Fig. 5E) and F 1s (588.68 eV; Fig. 5G) signals show intensity change tied to [TFSI] anion dynamics. However, identifying [EMIM] cations from the C 1s and N 1s spectra is difficult due to overlap with the polymer backbone. Instead, we infer their motion via the sulfur signal at 163.68 eV. Because XPS provides only the relative elemental composition within its sampling depth, it cannot tell us exactly where [EMIM] cations move during doping. Instead, we interpret the changes within the broader physical constraints of the system. During n-type doping, the clear decrease in the [TFSI] anion signal indicates anion depletion, while the accompanying decrease—not increase—in the 2Tz sulfur intensity suggests that this process is not simply exposing more of the polymer surface. Rather, the most consistent interpretation is that [EMIM] cations enter the film to compensate the injected electrons, maintaining electroneutrality without producing measurable swelling. This conclusion aligns with the combined operando evidence: GIWAXS shows minimal expansion of the lamellar spacing, AFM height maps show negligible topographical change, and EQCM-D detects <1% mass uptake. Similarly, during p-type doping, the substantial rise in the [TFSI] anion signal accompanied by only a modest change in the 2Tz sulfur signal is consistent with the partial expulsion of [EMIM] cations, which offsets the attenuation expected from anion accumulation. In addition, the S 2p spectra exhibit an increased intensity on the high–binding-energy side under p-type doping, indicating the formation of an oxidized sulfur environment. Because of the intrinsic spin-orbit splitting of the S 2p signal, this additional oxidized sulfur component partially overlaps with the original S 2p doublet, resulting in an apparent growth of the high binding energy peak rather than a distinct peak shift. These observations align well with DFT and EPR results, supporting the localization of polarons on the polymer. Overall, operando electrochemical XPS offers detailed insights into the redox behavior of the Tz and NDI units and the ion dynamics during electrochemical doping, as summarized schematically in Fig. 5I.

## DISCUSSION

In conclusion, this study provides a comprehensive investigation of the ambipolar character of P-6O under electrochemical doping, confirming the relative localization of polarons and the polymer’s lower planarity through a combination of operando electrochemical EPR, XPS, and DFT calculation. Temperature-dependent operando EPR and ATR-FTIR further reveal strong interactions between [EMIM][TFSI] and P-6O. Notably, morphological stability is maintained during doping, as evidenced by negligible swelling in operando GIWAXS and elastic modulus AFM, highlighting its importance for long-term device stability. Operando XPS studies offer additional insights into the ionic dynamics, showing how balanced ion transport preserves film volume during doping. Together, these results demonstrate that P-6O operates as an ambipolar organic mixed ionic-electronic conductor (OMIEC) in which localized electronic states, balanced ion transport, and suppressed volumetric deformation jointly enable stable OECT performance.

Beyond the material-specific observations, our operando results converge into a general mechanistic framework for OECT operation. In an OECT, the applied gate bias first drives ions from the electrolyte into (or out of) the polymer film and then induce the electronic charges on the polymer backbone (creating polarons or bipolarons), thereby maintain local electroneutrality. Thus, electronic charging and ionic motion form a tightly coupled, self-regulating process rather than two independent phenomena. Our operando XPS directly visualizes this coupled electronic-ionic process, while operando EPR and FTIR show how the evolving ionic environment modulates polaron energetics, demonstrating that ion redistribution and electronic localization are mutually reinforcing components of the charge-ion coupling that governs OECT behavior.

At the device level, operando GIWAXS, AFM, and EQCM-D reveal that this charge-ion coupling can proceed through a largely volume-preserving pathway, minimizing mechanical strain during repeated redox cycling. This finding contrasts with swelling-dominated OMIECs and highlights a broader design principle; OECT stability can also benefit from controlled ion-polymer interactions and suppressed volumetric deformation. These insights extend beyond P-6O and provide a general framework for engineering next-generation OECT materials that couple electronic and ionic transport in a mechanically robust manner.

## MATERIALS AND METHODS

### Materials

The synthesis details of D-A copolymer P-6O based on NDI-2Tz are described in the previous papers [(*M*_n_ = 105,000 and polydispersity index = 3.33) (*32*, *33*)]. Chloroform (CF) was purchased from Tianjin Kemio Chemical Reagent Co. Ltd. Isopropanol was purchased from Tianjin Damao Chemical Reagent Factory. Acetone was purchased from Tianjin Kemio Chemical Reagent Co. Ltd. PVDF-co-HFP was purchased from Shanghai McLean Biochemical Technology Co. Ltd. [EMIM][TFSI] was purchased from Shanghai Meirui Chemical Technology Co. Ltd. All chemicals are used as received.

### Methods

#### Device fabrication and characterizations

The silicon substrate was laser etched with a predesigned pattern at a power of 80 W and 35 Hz. Patterned silicon and glass substrates were sequentially treated with acetone, isopropanol, DECON90 cleaning (5%) solution, and deionized water by ultrasonic treatment for 20 min, followed by drying with N_2_, and then exposed to ultraviolet (UV) ozone for 30 min. Then, 30 nm of Cr and 200 nm of Au were deposited on a patterned silicon substrate and glass substrate using a template by the thermal evaporation method. Polymer was dissolved in CF at a concentration of 5 mg/ml. The composition of the gel electrolyte was acetone/PVDF-co-HFP/[EMIM][TFSI] with concentrations of 78/17.6/4.4 wt %.

#### Electrical characterization

The interdigitated microelectrodes were treated with UV ozone for 30 min; the specific parameters of interdigitated microelectrodes are summarized in the Supplementary Materials). Following spin coating of the polymer solutions at 1000 rpm for 30 s, they were heated at 110°h for 20 min on the heating table for annealing treatment. Electrical characteristics of OECT were measured by a Keithley 2636B source meter with KickStart software. The electrolyte was dropped onto the P-6O film, and Ag/AgCl pellet was used as the gate. The stability test of the device was measured in a nitrogen atmosphere.

#### Operando ultraviolet–visible–near infrared spectroscopy

The ultraviolet-visible-near infrared absorption spectra were measured by UH5700 (Hitachi) spectrophotometer. The scanning wavelength was from 300 to 1500 nm, and a temperature controller cell was used for temperature-dependent measurement. First, the polymer solutions were spin coated at 1000 rpm for 30 s onto clean ITO glass. Then, the film was heated at 110°C for 20 min on a heating table for annealing treatment. Subsequently, a gel electrolyte was dropcast on top of the polymer film, and an Ag/AgCl pellet was used as the gate electrode. To prevent the electrolyte crystallization, the sample was heated at 110°C for 20 min and then fast cooled with N_2_ flow over the ITO. When the gate is connected to the positive electrode of the power supply, the polymer exhibits n-type semiconductor properties; conversely, it exhibits p-type semiconductor properties. This convention applies to all subsequent measurements.

#### Operando EPR

A polymer film was drop coated onto a clean PET substrate with gold electrode. The gel electrolyte was dripped onto the film, and an Ag/AgCl pellet was placed on the electrolyte to serve as the gate electrode. The devices were placed in a cylindrical quartz tube and inserted into the resonant position of the EPR device (EPR200M, CIQTEK). Following each voltage application, a 5-min stabilization period was observed before performing the test. This procedure was followed consistently. Each test was carried out for 4 cycles to obtain the final results.

#### Operando XPS

An x-ray photoelectron spectrometer (ESCALAB 250Xi, Thermo Fisher Scientific, USA) was used for this experiment. The analyzing chamber had a vacuum of 8 × 10^−10^ mbar. The excitation source was Al Kα rays (*hv* = 1486.6 eV), with an operating voltage of 12.5 kV and a filament current of 16 mA. Signal accumulation was performed for 5 to 10 cycles. The pass energy was 40 eV with a step of 0.1 eV. Operando XPS spectra were collected on the same device following the sequence n-doping → reset to 0 V → p-doping. Gate biases were applied during XPS acquisition to maintain the corresponding doping states. After each measurement, the device was held at the next bias for 2 min to ensure full electrochemical equilibration. After the n-doping measurement, the device was returned to 0 V and then subjected to p-doping. Charge correction was performed using the C 1s peak at 284.80-eV binding energy as a reference.

#### Operando AFM

Operando AFM measurements were conducted on a Bruker Dimension Icon microscope using the PeakForce QNM mode. A silicon RFESP-75 probe with a nominal spring constant of *k* = 3 N/m was used. Topography and elastic modulus maps were acquired synchronously over a scan area of 10 μm by 10 μm. The device architecture mirrored that used for operando GIWAXS measurements, with the gate voltage controlled by a Keithley 2450 source meter. The testing protocol involved stepping the gate voltage from 0 to +1.2 V in 0.3 V increments, followed by a return to 0 V. Subsequently, the probe was relocated to a pristine area on the device, where a reverse voltage sequence from 0 to −1.2 V and back to 0 V was applied.

#### Operando GIWAXS

The GIWAXS data were obtained at 1W1A Diffuse X-ray Scattering Station, Beijing Synchrotron Radiation Facility (BSRF-1W1A). The monochromatic of the light source was 1.54 Å. The data were recorded using the 2D image plate detector of EIGER lM from Dectris, Switzerland. The total exposure time measured for all samples was 30 s, and the incident angle of the light source during the testing process was 0.2°.

#### Electrochemical Quartz Crystal Microbalance with Dissipation

EQCM-D was conducted using a Q-Sense analyzer (QE401, Q-sense AB, Biolin Scientific) coupled with a PguTouch electrochemical workstation (IPS Elektroniklabor GmbH & Co. KG). Gold-coated quartz crystal sensors (QSX 338, 5 MHz, Biolin Scientific) served as the substrate.

To accurately quantify the polymer mass and passive swelling, a background correction protocol was used. First, the resonance frequency (*f*) and dissipation (*D*) of the clean, bare Au sensors were recorded in air and subsequently in the electrolyte (0.1 M [EMIM][TFSI] in acetonitrile). This step established a baseline to decouple the viscous loading effects caused by the liquid medium’s density and viscosity. The sensors were then removed, dried, and spin coated with the P-6O active layer from a solution (5 mg/ml) at 1000 rpm. The coated sensors were remounted, and their signals were recorded again in both air and the electrolyte. 

In situ electrochemical measurements were performed in a flow module using a two-electrode configuration, where the polymer-coated sensor acted as the working electrode and a platinum wire served as the counter electrode (also functioning as a pseudoreference). The mass change (Δ*m*) was calculated from the frequency shift (Δ*f_n_*) of the selected overtones (*n* = 3) using the Sauerbrey equation

$$∆m=-\frac{C\cdot ∆f_{n}}{n}$$

where *C* is the mass sensitivity constant (17.7 ng cm^−2^ Hz^−1^ for 5-MHz crystals). The dissipation factor was monitored throughout the experiment to ensure the validity of the rigid-film assumption.

## References

[1] D. A. Bernards, G. G. Malliaras, Steady-state and transient behavior of organic electrochemical transistors. Adv. Funct. Mater. 17, 3538–3544 (2007).

[2] W. Huang, J. Chen, Y. Yao, D. Zheng, X. Ji, L. W. Feng, D. Moore, N. R. Glavin, M. Xie, Y. Chen, R. M. Pankow, A. Surendran, Z. Wang, Y. Xia, L. Bai, J. Rivnay, J. Ping, X. Guo, Y. Cheng, T. J. Marks, A. Facchetti, Vertical organic electrochemical transistors for complementary circuits. Nature 613, 496–502 (2023). 36653571 10.1038/s41586-022-05592-2 PMC9849123

[3] A. Nawaz, Q. Liu, W. L. Leong, K. E. Fairfull-Smith, P. Sonar, Organic electrochemical transistors for in vivo bioelectronics. Adv. Mater. 33, e2101874 (2021). 34606146 10.1002/adma.202101874

[4] K. Guo, S. Wustoni, A. Koklu, E. Diaz-Galicia, M. Moser, A. Hama, A. A. Alqahtani, A. N. Ahmad, F. S. Alhamlan, M. Shuaib, A. Pain, I. McCulloch, S. T. Arold, R. Grunberg, S. Inal, Rapid single-molecule detection of COVID-19 and MERS antigens via nanobody-functionalized organic electrochemical transistors. Nat. Biomed. Eng. 5, 666–677 (2021). 34031558 10.1038/s41551-021-00734-9

[5] B. D. Paulsen, K. Tybrandt, E. Stavrinidou, J. Rivnay, Organic mixed ionic-electronic conductors. Nat. Mater. 19, 13–26 (2020). 31427743 10.1038/s41563-019-0435-z

[6] N. A. Kukhta, A. Marks, C. K. Luscombe, Molecular design strategies toward improvement of charge injection and ionic conduction in organic mixed ionic-electronic conductors for organic electrochemical transistors. Chem. Rev. 122, 4325–4355 (2022). 34902244 10.1021/acs.chemrev.1c00266 PMC8874907

[7] H. Y. Wu, J. D. Huang, S. Y. Jeong, T. Liu, Z. Wu, T. van der Pol, Q. Wang, M. A. Stoeckel, Q. Li, M. Fahlman, D. Tu, H. Y. Woo, C. Y. Yang, S. Fabiano, Stable organic electrochemical neurons based on p-type and n-type ladder polymers. Mater. Horiz. 10, 4213–4223 (2023). 37477499 10.1039/d3mh00858d

[8] S. T. Keene, J. E. M. Laulainen, R. Pandya, M. Moser, C. Schnedermann, P. A. Midgley, I. McCulloch, A. Rao, G. G. Malliaras, Hole-limited electrochemical doping in conjugated polymers. Nat. Mater. 22, 1121–1127 (2023). 37414944 10.1038/s41563-023-01601-5 PMC10465356

[9] B. D. Paulsen, A. Giovannitti, R. Wu, J. Strzalka, Q. Zhang, J. Rivnay, C. J. Takacs, Electrochemistry of thin films with in situ/operando grazing incidence x-ray scattering: Bypassing electrolyte scattering for high fidelity time resolved studies. Small 17, e2103213 (2021). 34549509 10.1002/smll.202103213

[10] D. Ohayon, V. Druet, S. Inal, A guide for the characterization of organic electrochemical transistors and channel materials. Chem. Soc. Rev. 52, 1001–1023 (2023). 36637165 10.1039/d2cs00920j

[11] T. Pan, X. Jiang, E. R. W. van Doremaele, J. Li, T. P. A. van der Pol, C. Yan, G. Ye, J. Liu, W. Hong, R. C. Chiechi, Y. V. de Burgt, Y. Zhang, Over 60 h of stable water-operation for n-type organic electrochemical transistors with fast response and ambipolarity. Adv. Sci. 11, 2400872 (2024). 10.1002/advs.202400872 PMC11304290 38810112

[12] Y. Zhang, E. R. W. van Doremaele, G. Ye, T. Stevens, J. Song, R. C. Chiechi, Y. van de Burgt, Adaptive biosensing and neuromorphic classification based on an ambipolar organic mixed ionic–electronic conductor. Adv. Mater. 34, e2200393 (2022). 35334499 10.1002/adma.202200393

[13] G. Ye, J. Liu, X. Qiu, S. Stater, L. Qiu, Y. Liu, X. Yang, R. Hildner, L. J. A. Koster, R. C. Chiechi, Controlling n-type molecular doping via regiochemistry and polarity of pendant groups on low band gap donor-acceptor copolymers. Macromolecules 54, 3886–3896 (2021). 34054145 10.1021/acs.macromol.1c00317 PMC8154869

[14] J. Liu, G. Ye, B. V. Zee, J. Dong, X. Qiu, Y. Liu, G. Portale, R. C. Chiechi, L. J. A. Koster, n-type organic thermoelectrics of donor-acceptor copolymers: Improved power factor by molecular tailoring of the density of states. Adv. Mater. 30, e1804290 (2018). 30222216 10.1002/adma.201804290

[15] Z. Wang, H. Wang, M. Du, X. Lai, F. He, Q. Guo, Q. Guo, A. Tang, X. Sun, E. Zhou, Naphthodithiophene diimide (NDTI)-based cathode interlayer material enables 19% efficiency binary organic solar cells. Adv. Funct. Mater. 34, 2313240 (2023).

[16] P. K. Kahol, N. J. Pinto, An EPR investigation of electrospun polyaniline-polyethylene oxide blends. Synth. Met. 140, 269–272 (2004).

[17] T. Tibaoui, S. Ayachi, M. Hamidi, M. Bouachrine, M. Paris, K. Alimi, Characterization and structure–property relationship of chemical oxidative polymerization of poly(para-hydroquinone). J. Appl. Polym. Sci. 118, 711–720 (2010).

[18] W. Domagala, B. Pilawa, M. Lapkowski, Quantitative in-situ EPR spectroelectrochemical studies of doping processes in poly(3,4-alkylenedioxythiophene)s. Electrochim. Acta 53, 4580–4590 (2008).

[19] Y. Lu, Z. D. Yu, H. I. Un, Z. F. Yao, H. Y. You, W. Jin, L. Li, Z. Y. Wang, B. W. Dong, S. Barlow, E. Longhi, C. A. Di, D. Zhu, J. Y. Wang, C. Silva, S. R. Marder, J. Pei, Persistent conjugated backbone and disordered lamellar packing impart polymers with efficient n-doping and high conductivities. Adv. Mater. 33, e2005946 (2021). 33251668 10.1002/adma.202005946

[20] A. Privitera, R. Warren, G. Londi, P. Kaienburg, J. Liu, A. Sperlich, A. E. Lauritzen, O. Thimm, A. Ardavan, D. Beljonne, M. Riede, Electron spin as fingerprint for charge generation and transport in doped organic semiconductors. J. Mater. Chem. C 9, 2944–2954 (2021).

[21] L. M. Bongartz, R. Kantelberg, T. Meier, R. Hoffmann, C. Matthus, A. Weissbach, M. Cucchi, H. Kleemann, K. Leo, Bistable organic electrochemical transistors: Enthalpy vs. entropy. Nat. Commun. 15, 6819 (2024). 39122689 10.1038/s41467-024-51001-9 PMC11316041

[22] B. Asbani, B. Bounor, K. Robert, C. Douard, L. Athouël, C. Lethien, J. Le Bideau, T. Brousse, Reflow soldering-resistant solid-state 3D micro-supercapacitors based on ionogel electrolyte for powering the internet of things. J. Electrochem. Soc. 167, 105609 (2020).

[23] I. Denti, S. Cimò, L. Brambilla, A. Milani, C. Bertarelli, M. Tommasini, C. Castiglioni, Polaron confinement in n-doped P(NDI2OD-T2) unveiled by vibrational spectroscopy. Chem. Mater. 31, 6726–6739 (2019).

[24] J. F. Kiefer, A. Leipertz, Experimental vibrational study of imidazolium-based ionic liquids: Raman and infrared spectra of 1-ethyl-3-methylimidazolium bis(trifluoromethylsulfonyl)imide and 1-ethyl-3-methylimidazolium ethylsulfate. Appl. Spectrosc. 61, 1306–1311 (2007). 18198022 10.1366/000370207783292000

[25] R. Brooke, M. Fabretto, M. Krasowska, P. Talemi, S. Pering, P. J. Murphy, D. Evans, Organic energy devices from ionic liquids and conducting polymers. J. Mater. Chem. C 4, 1550–1556 (2016).

[26] V. Armel, J. Rivnay, G. Malliaras, B. Winther-Jensen, Unexpected interaction between PEDOT and phosphonium ionic liquids. J. Am. Chem. Soc. 135, 11309–11313 (2013). 23834210 10.1021/ja405032c

[27] K. Fumino, R. Ludwig, Analyzing the interaction energies between cation and anion in ionic liquids: The subtle balance between Coulomb forces and hydrogen bonding. J. Mol. Liq. 192, 94–102 (2014).

[28] H. Zheng, Y. Liu, H. Ma, Y. Wang, G. Zhou, Z. Cao, K. Xu, In-situ electrochemical doping of poly(3-hexylthiophene) with insights into interfacial water molecule dynamics and film swelling contributions. Polymer 321, 128089 (2025).

[29] Y. Zhang, G. Ye, T. P. A. van der Pol, J. Dong, E. R. W. van Doremaele, I. Krauhausen, Y. Liu, P. Gkoupidenis, G. Portale, J. Song, R. C. Chiechi, Y. van de Burgt, High-performance organic electrochemical transistors and neuromorphic devices comprising naphthalenediimide-dialkoxybithiazole copolymers bearing glycol ether pendant groups. Adv. Funct. Mater. 32, 2201593 (2022).

[30] C. G. Bischak, L. Q. Flagg, K. Yan, T. Rehman, D. W. Davies, R. J. Quezada, J. W. Onorato, C. K. Luscombe, Y. Diao, C. Z. Li, D. S. Ginger, A reversible structural phase transition by electrochemically-driven ion injection into a conjugated polymer. J. Am. Chem. Soc. 142, 7434–7442 (2020). 32227841 10.1021/jacs.9b12769

[31] B. D. Paulsen, R. Wu, C. J. Takacs, H. G. Steinruck, J. Strzalka, Q. Zhang, M. F. Toney, J. Rivnay, Time-resolved structural kinetics of an organic mixed ionic-electronic conductor. Adv. Mater. 32, e2003404 (2020). 32864811 10.1002/adma.202003404

[32] X. Yang, G. Ye, K. Tran, Y. Liu, J. Cao, J. Dong, G. Portale, J. Liu, P. Zhang, M. A. Loi, R. C. Chiechi, L. J. A. Koster, Impact of oligo(ethylene glycol) side chains on the thermoelectric properties of naphthalenediimide–dialkoxybithiazole polymers. ACS Mater. Lett. 6, 1207–1215 (2024).

[33] J. Liu, G. Ye, H. G. O. Potgieser, M. Koopmans, S. Sami, M. I. Nugraha, D. R. Villalva, H. Sun, J. Dong, X. Yang, X. Qiu, C. Yao, G. Portale, S. Fabiano, T. D. Anthopoulos, D. Baran, R. W. A. Havenith, R. C. Chiechi, L. J. A. Koster, Amphipathic side chain of a conjugated polymer optimizes dopant location toward efficient n-type organic thermoelectrics. Adv. Mater. 33, e2006694 (2021). 33306230 10.1002/adma.202006694 PMC11468643

