# Supplementary material for: Multimodal operando characterization unravels polaron accumulation and ion dynamics in high-stability ambipolar OECTs
Source: Sci Adv. 2026 Apr 15;12(16):eaea9786. doi: 10.1126/sciadv.aea9786 (PMC13082333; doi:10.1126/sciadv.aea9786)
Supplement: Supplementary file 1 — Figs. S1 to S17 [file sciadv.aea9786_sm.pdf]

Science Advances
AAAS

Supplementary Materials for

Multimodal operando characterization unravels polaron accumulation and ion dynamics in high-stability ambipolar OECTs

Haoyu Zheng *et al.*

Corresponding author: Gang Ye, g.ye0612@hubu.edu.cn; Gang Wang, gwf8707@dhu.edu.cn; Hengda Sun, sunhengda@dhu.edu.cn; Kai Xu, xukai@ysu.edu.cn

Sci. Adv. **12**, eaea9786 (2026)
DOI: 10.1126/sciadv.aea9786

This PDF file includes:

Figs. S1 to S17

Supplementary Figures

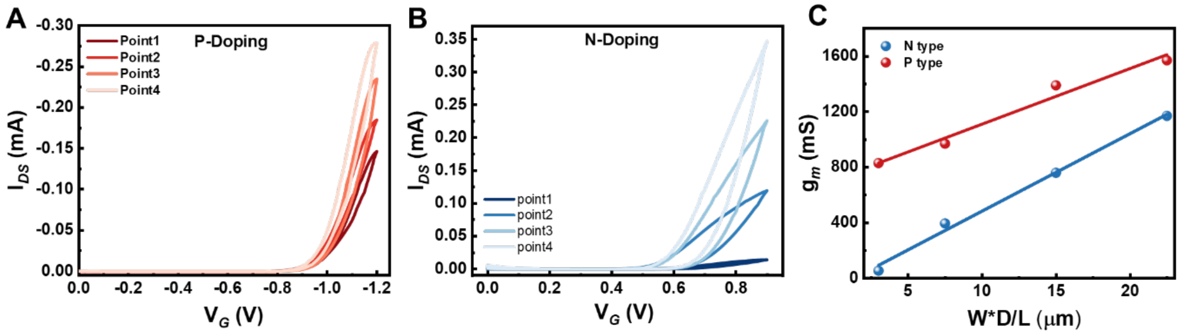

Fig. S1. Channel-dimension-dependent transfer characteristics and extracted μC* of ambipolar OECTs. Transfer curves for p-type doping (**A**) and n-type doping (**B**) with varying channel dimensions. The channel widths are 0.18 cm at point 1, 0.45 cm at point 2, 0.90 cm at point 3, and 1.35 cm at point 4, with a channel length of 30 μm and a film thickness of 50 nm, the derived V_{th} of p-doping is about -0.9 V, and 0.5 V for n-type doping. (**C**) Peak transconductances as a function of active layer dimensionality (W*d/L), the voltage obtained at maximum transconductance for p-type doping is about -1.1 V, and 0.7 V for n-type doping, along with relative linear fitting showing μC^* of 2.8 F V^{-1} cm^{-1} S^{-1} for n-type and 2.0 F V^{-1} cm^{-1} S^{-1} for p-type doping.

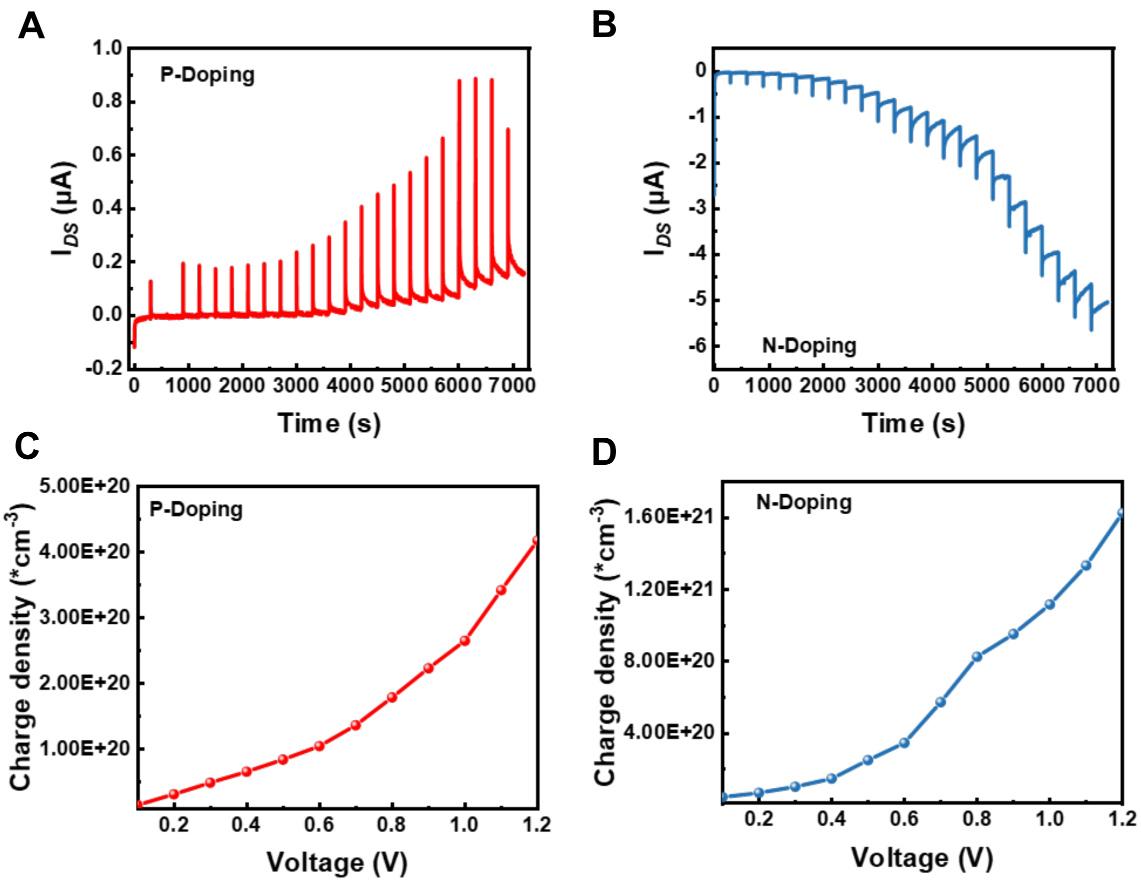

Fig. S2. Stepwise electrochemical charging of the polymer film in a two-electrode configuration. Pulse step voltage for charging the polymer film in a two-electrode setup. The polymer was deposited on an Au substrate, covered with an electrolyte and connected to a sintered Ag/AgCl pellet. The voltage was stepped from 0 V to 1.2 V in increments of 0.05 V. (**A**) P-type charging current and (**B**) n-type charging current, with the integrated charge density shown in (**C**) and (**D**).

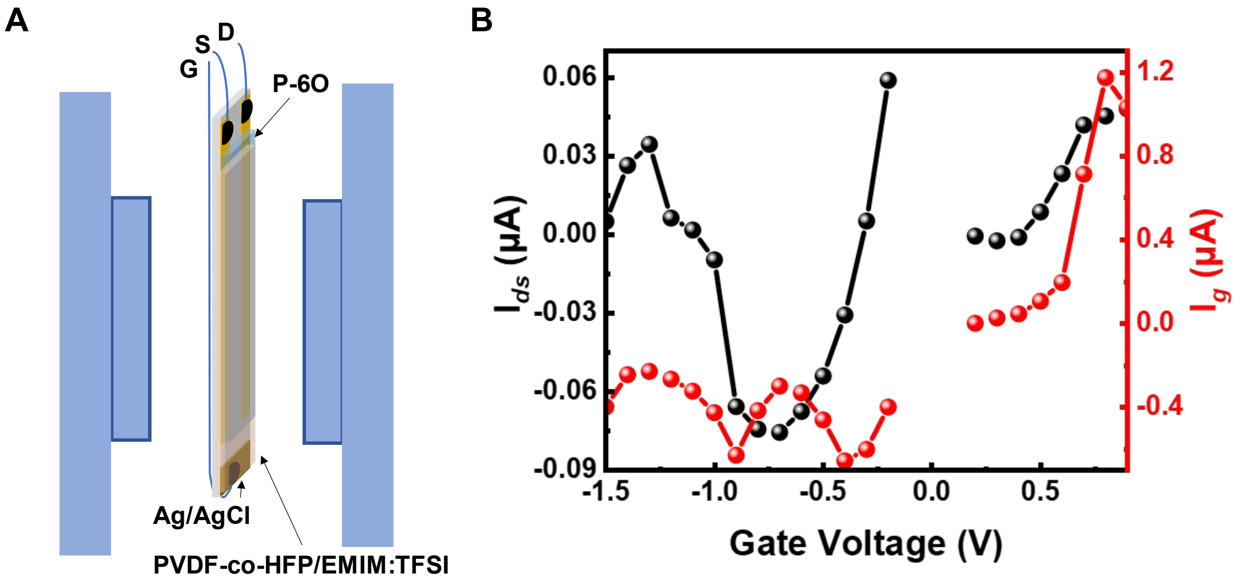

Fig. S3. Operando EPR measurement setup and corresponding electrical characteristics. (**A**) Schematic of the device for in-situ EPR test. (**B**) The corresponding gate current (red triangle) and drain current (black square) under different gate voltages, with V_{SD}=0.1 V under N-type doping, and -0.1 V under P-type doping.

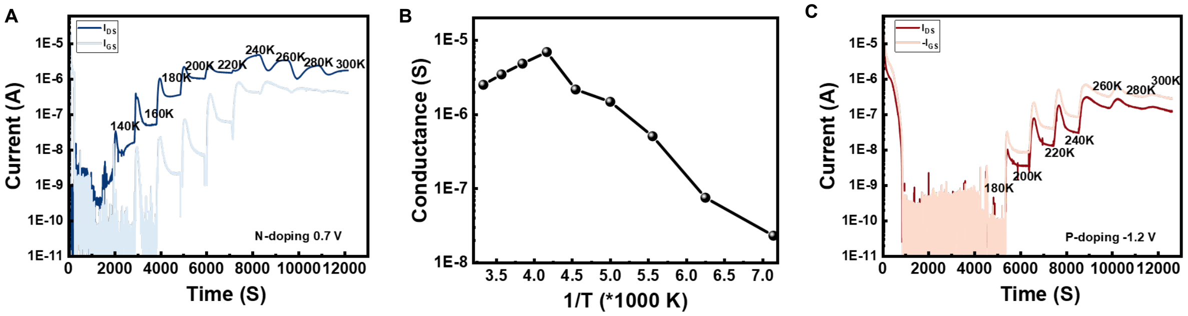

Fig. S4. Temperature-dependent electrical response during operando EPR measurements under fixed electrochemical doping conditions. The obtained gate and drain current during in-situ EPR test of N type doping at 0.7 V (**A**), and P type doping at -1.2 V (**C**), (**B**) the extracted conductance of N doping as a function of temperature, the decrease in conductance at high temperatures may be attributed to entropy dominance as the temperature rises (*31*).

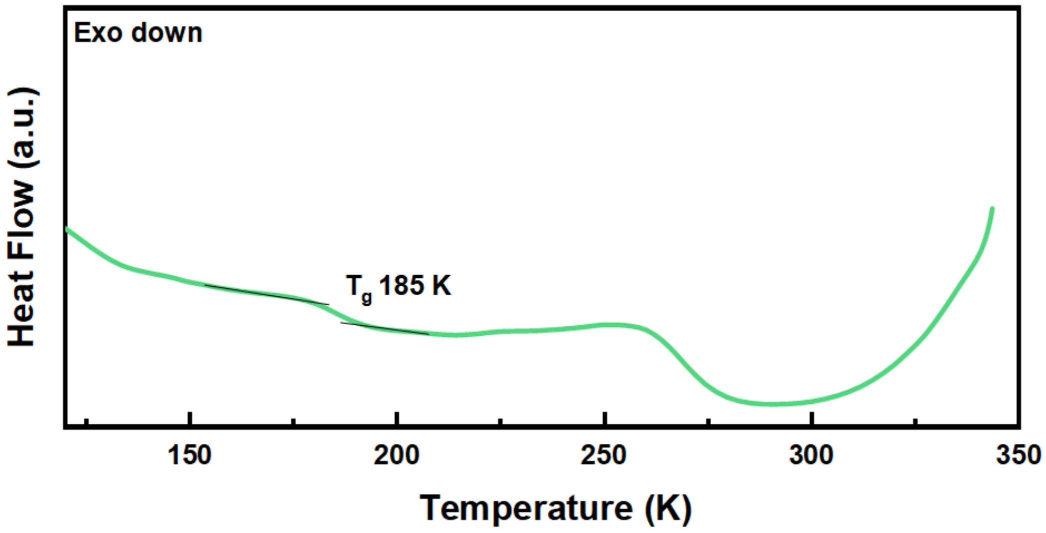

Fig. S5. Thermal transitions of the PVDF-co-HFP/EMIM:TFSI ion gel characterized by DSC. DSC measurements of the PVDF-co-HFP/EMIM:TFSI ion gel.

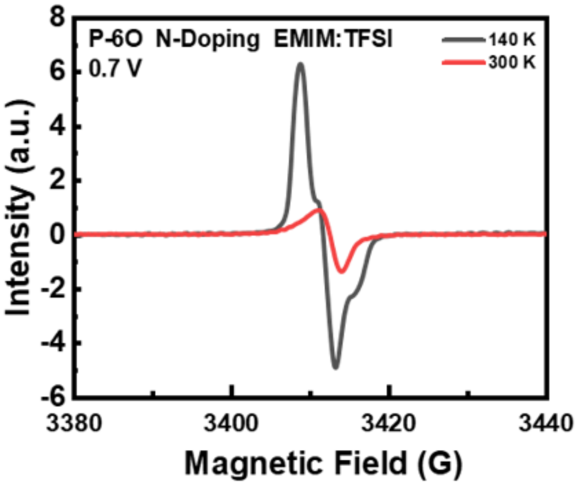

Fig. S6. Temperature-dependent operando EPR spectra of n-type doped P-6O in EMIM:TFSI. Comparison of N-Doping in P-6O with [EMIM][TFSI] at different temperatures.

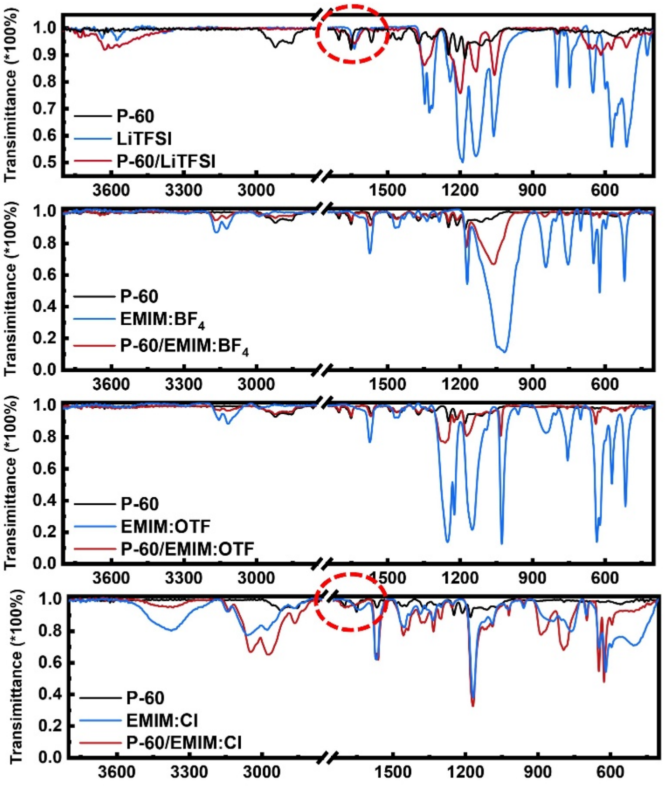

Fig. S7. ATR-FTIR evidence of polymer–electrolyte interactions in different ionic liquids. ATR-FTIR spectra of pristine P-6O and four different ionic liquids (LiTFSI, $EMIMBF_4$, EMIMOTf, and EMIMCl). For each ionic liquid, the spectrum of a P-6O film in contact with that ionic liquid (green line) is shown in the corresponding panel. Notably, the backbone vibrational modes of P-6O (1700 cm^{-1} and 1648 cm^{-1}) exhibit clear distortions when in contact with LiTFSI and EMIMCl.

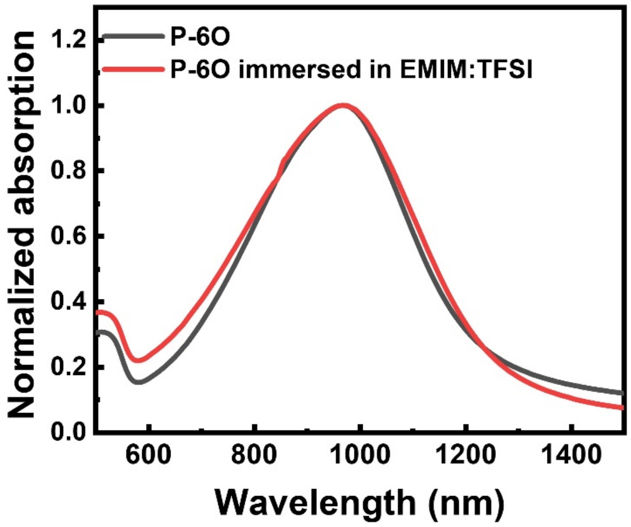

Fig. S8. UV–vis absorption spectra of P-6O before and after contact with EMIM:TFSI. UV–vis absorption spectra of pristine P-6O and P-6O films immersion in EMIM: TFSI, showing the broadening of the polymer absorption upon contact with the ionic liquid.

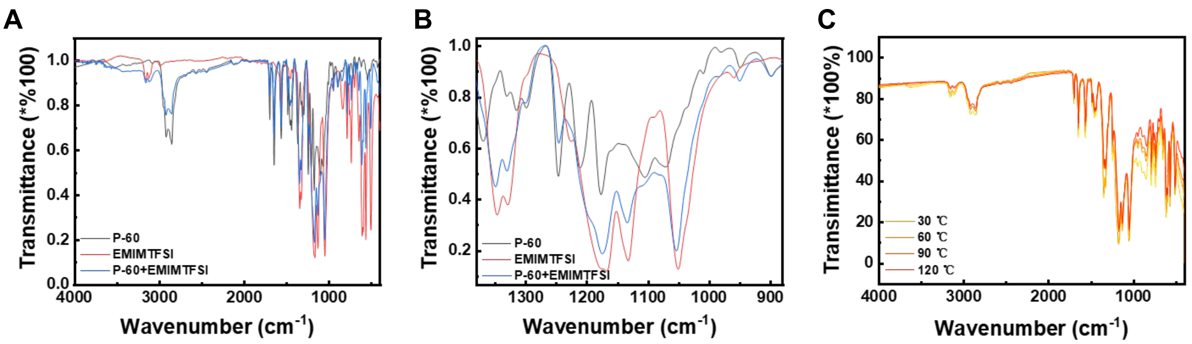

Fig. S9. ATR-FTIR analysis of pristine components and P-6O/EMIM:TFSI composite films. (**A**) Full ATR-FTIR spectrum from 4000 cm^{-1} to 400 cm^{-1} for pristine P-6O, [EMIM][TFSI], and the P-6O/[EMIM][TFSI] composite film. (**B**) Enlarged spectrum in the range of 1400 cm^{-1} to 900 cm^{-1}. (**C**) Full ATR-FTIR spectrum from 4000 cm^{-1} to 400 cm^{-1} of the composite film at different temperatures.

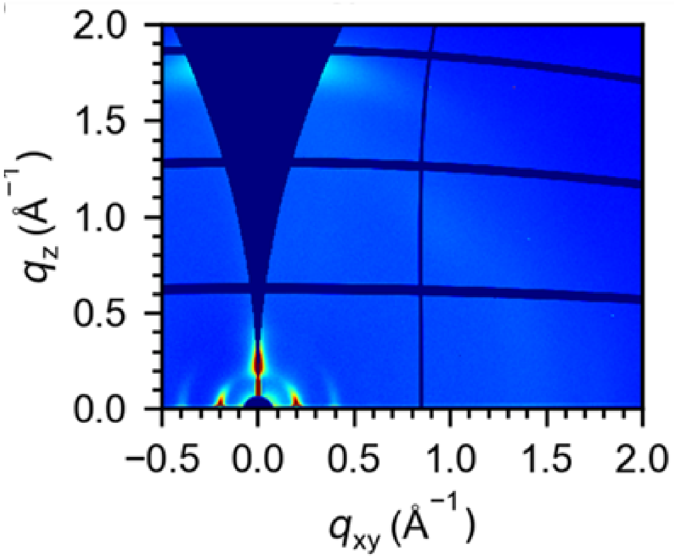

Fig. S10. Two-dimensional GIWAXS patterns of pristine P-6O. 2D GIWAXS of pristine P-6O film. The GIWAXS data were obtained at BL02U2, Shanghai Synchrotron Radiation Facility. The total exposure time measured for sample was 5 s, and the incident angle of the light source during the testing process was 0.2° .

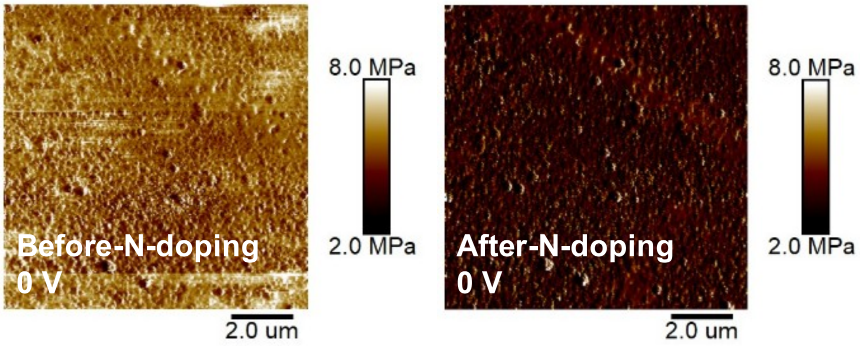

Fig. S11. AFM modulus maps of the polymer film before and after electrochemical doping. Atomic force microscopy (AFM) modulus maps of the polymer film measured at 0 V before and after completing the n-doping.

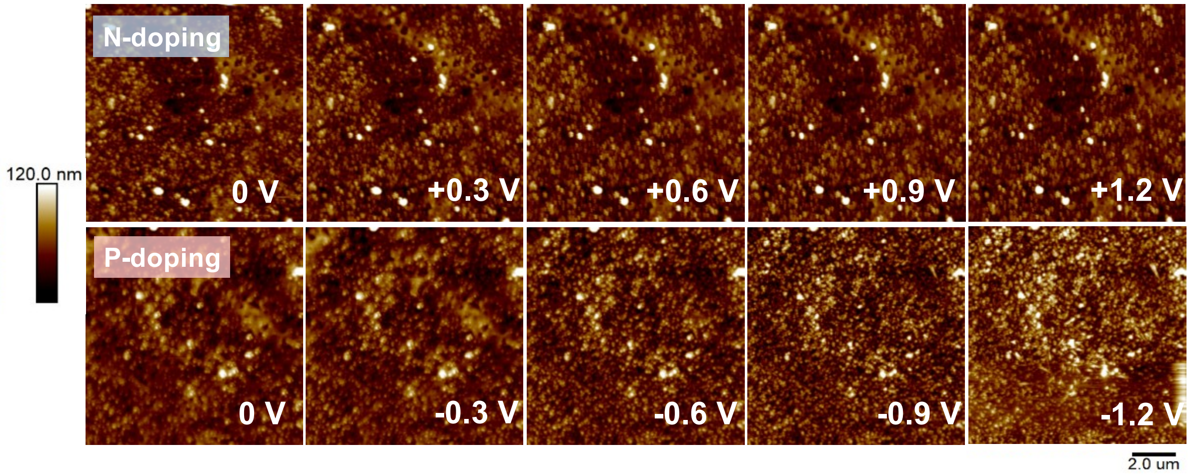

Fig. S12. Surface morphology of the polymer film under electrochemical n-type and p-type doping. AFM height maps of the polymer film under n-type and p-type doping.

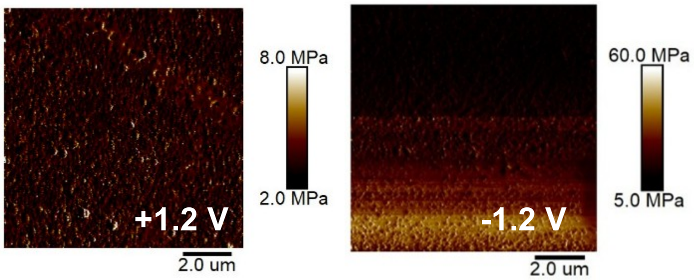

Fig. S13. AFM modulus maps of the polymer film during electrochemical doping. AFM modulus maps of the polymer film measured at +1.2 V and -1.2 V.

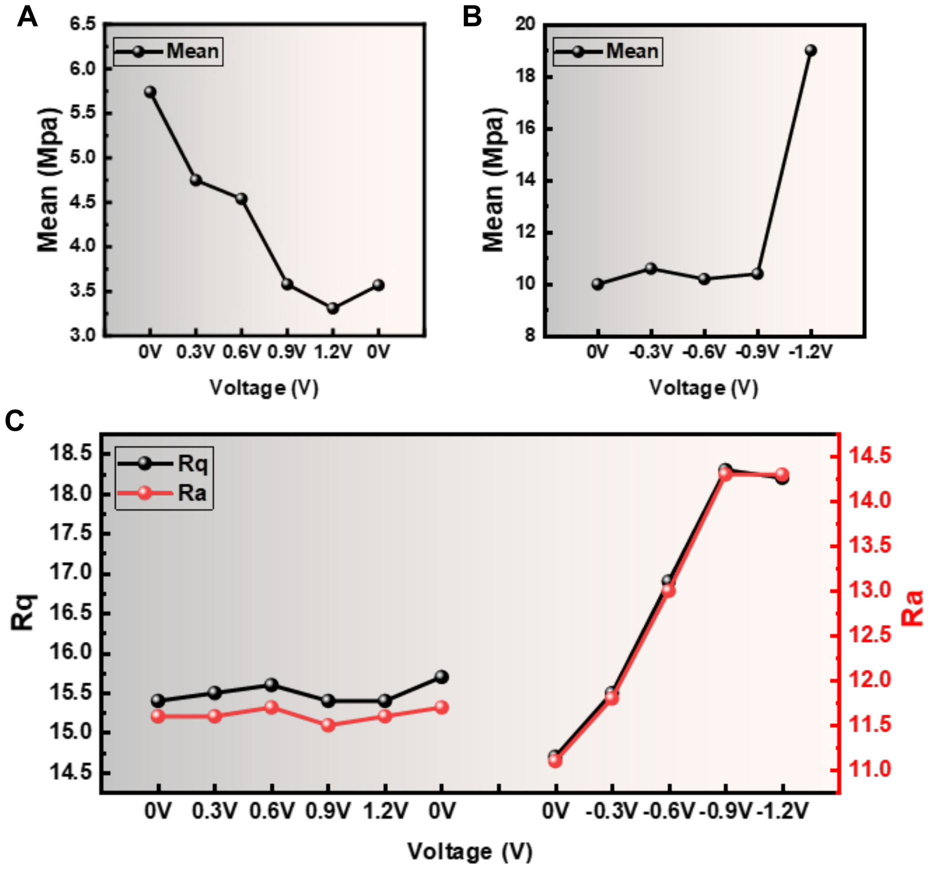

Fig. S14. Comparison of elastic modulus and surface roughness under electrochemical doping. Extracted mean elastic modulus (**A, B**) and surface roughness (**C**) of the polymer film under n-type and p-type doping conditions.

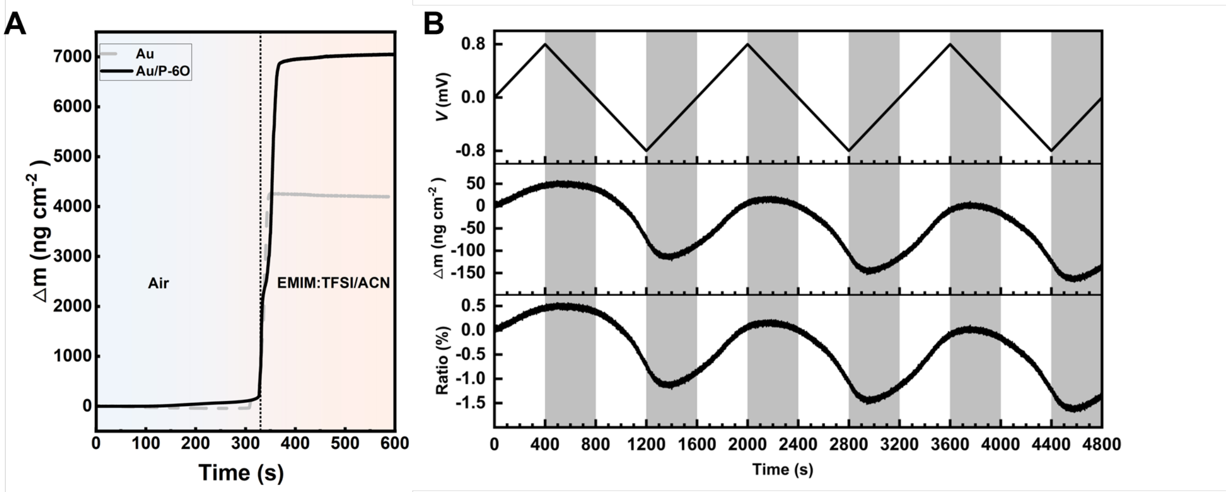

Fig. S15. Mass uptake and electrochemical response of P-6O films in EMIM:TFSI measured by EQCM-D. Electrochemical quartz crystal microbalance with dissipation monitoring (EQCM-D) measurements of a P-6O film in [EMIM][TFSI] solution. P-6O was coated on a gold crystal with an initial mass of 7227 ng/cm². (**A**) Mass changes of the P-6O film and a pristine Au crystal upon contact with the electrolyte, where the relative mass increase reflects electrolyte uptake by the film. (**B**) Cyclic voltammetry performed in the EQCM-D cell at a scan rate of 2 mV/s.

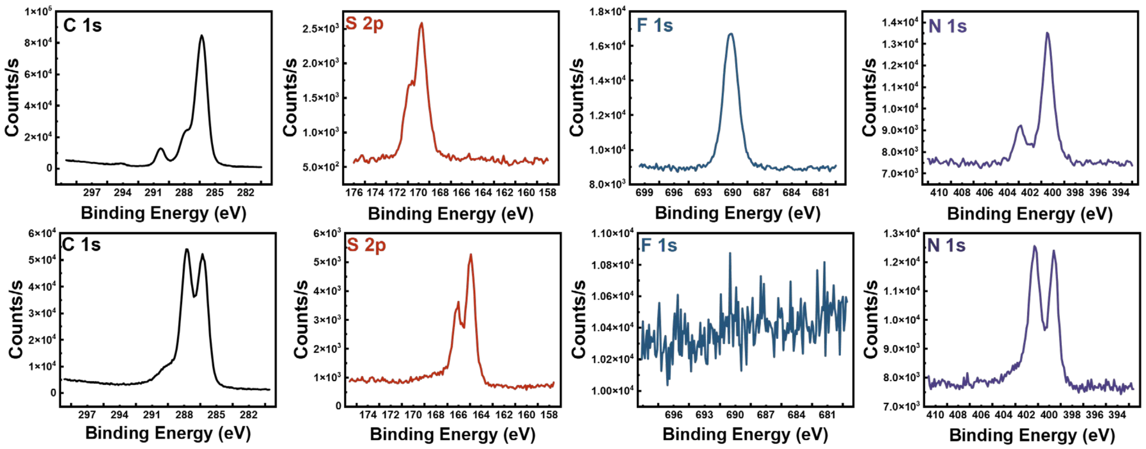

Fig. S16. XPS spectra of pristine EMIM:TFSI and pristine P-6O. Detailed C1s, S2p, F1s, and N1s spectra of pristine [EMIM][TFSI] (top panels) and pristine P-6O (bottom panels).

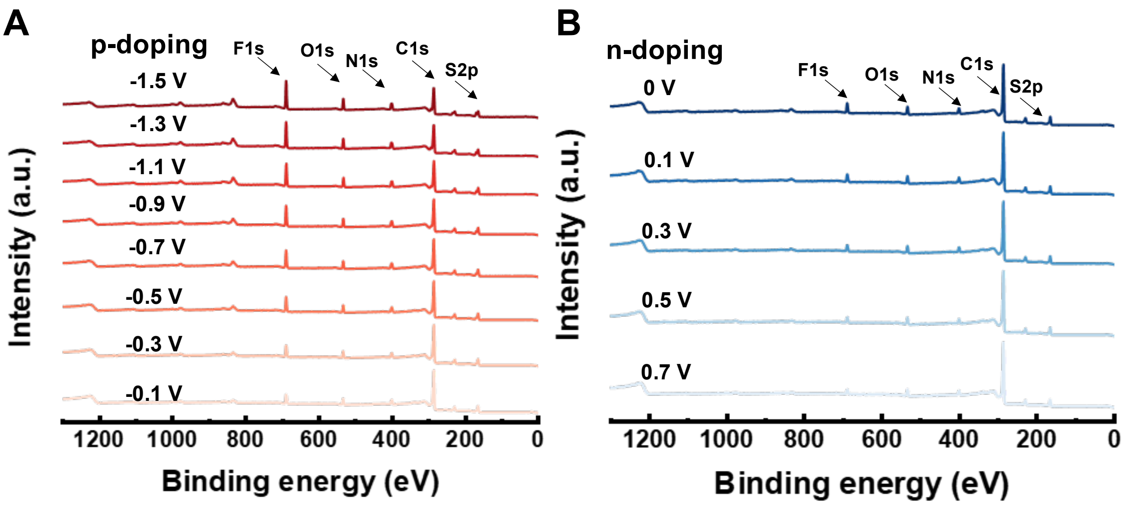

Fig. S17. Full XPS spectra of P-6O films under electrochemical p-type and n-type doping. (**A**) p-type doping and (**B**) n-type doping.
